# Challenges to reduce the ‘10/90 gap’: mental health research in Latin American and Caribbean countries

**DOI:** 10.1111/j.1600-0447.2008.01242.x

**Published:** 2008-12

**Authors:** D Razzouk, C Gallo, S Olifson, R Zorzetto, F Fiestas, G Poletti, G Mazzotti, I Levav, J J Mari

**Affiliations:** 1 Department of Psychiatry, Federal University of Sao Paulo (UNIFESP)São Paulo, Brazil; 2 Laboratory of Research and Development, Faculty of Sciences and Philosophy, University Peruana Cayetano HerediaLima, Peru; 3 Research and Programmes, Global Forum for Health ResearchGeneva, Switzerland; 4 Mental Health Services, Ministry of HealthJerusalem, Israel

**Keywords:** Latin America, Caribbean region, mental health, psychiatry, developing countries

## Abstract

Razzouk D, Gallo C, Olifson S, Zorzetto R, Fiestas F, Poletti G, Mazzotti G, Levav I, Mari JJ. Challenges to reduce the ‘10/90 gap’: mental health research in Latin American and Caribbean countries.

**Objective:**

To analyze the status of mental health research in 30 Latin American and Caribbean countries (LAC).

**Method:**

Medline and PsycInfo databases were searched to identify the LAC authors. Their publications were classified according to the topic, type of research and target population studied. Scientific indicators of these countries were assessed in other two different databases: Essential Scientific Information and Atlas of Science Project, both from Institute for Scientific Information.

**Results:**

Indexed-publications were concentrated in six countries: Argentina, Brazil, Chile, Colombia, Mexico and Venezuela. Most studies dealt with the burdensome mental disorders but neglected important topics such as violence and other mental health priorities.

**Conclusion:**

Mental health research is mostly concentrated in a few LAC countries, but these countries would contribute to reduce the research gap, if they provide research training to their neighbors and engage in bi- or multi-lateral research collaboration on common region priorities.

**Significant outcomes**

- Mental health research is concentrated in six countries: Argentina, Brazil, Chile, Colombia, Mexico and Venezuela and one-fourth of LAC countries had no mental health publication in all databases assessed in this study.
- Burdensome conditions such as violence, suicide, dementia and childhood disorders are under researched, as well as vulnerable population such as disabled, elderly, women, children and persons exposed to violence.
- There is a scarcity of studies on mental health systems and policies, clinical trials, epidemiology and cost-effectiveness of interventions. Low production of knowledge addressed to reduce the burden of mental disorders and to inform decision-makers.

**Limitations**

- Mental health research was not assessed in local and regional databases in where most of LAC countries’ output is published.
- The analysis of indexed-publications (Medline and PsycInfo) and the scientific indicators available in ISI databases were assessed in different periods, and the search developed for mental health studies in Medline and PsycInfo databases probably covered different domains of those included in the category ‘Psychiatry and Psychology’ provided by ISI databases. It might be possible that some papers related with mental health were not correctly indexed in Psychiatry/Psychology category according to ISI (Essential Science Indicators database) classification.
- Only the first authors were counted to identify the number of researchers by country in Medline/PsycInfo publications and this number may be underestimated.

## Introduction

Mental disorders account for 13% of the global burden of disease, and yet mental health-related publications amounted to less than 4% of the global health literature in the Institute for Scientific Information (ISI) database from 1992 to 2001 (1–4). Mental health research is even more neglected in low-and middle-income (LAMI) countries where mental disorders account for an important fraction of the burden of disease (1–13). While in Latin American and Caribbean (LAC) countries the burden of neuropsychiatric disorders is rising (1, 4, 12–17), mental health research related to burden issues remains poorly funded. Indeed, most governments allocate less than 1% of their gross domestic product (GDP) to research and human development, and less than 1% of their health budget to mental health (14, 18–22). Of all LAMI countries, those in LAC region had the lowest percentage of MEDLINE journal articles devoted to non-communicable diseases between 1998 and 2003 (11). In addition, LAC countries were less represented than other LAMI countries in the leading psychiatric journals (10). This uneven representation has improved in recent years: LAC countries have shown a rise in the number of scientific publications in journals indexed in Medline and ISI, a growth in the number of master's and doctoral students, and a modest increase in research funding (18, 23–34). In 1990, the Commission on Health Research for Development estimated that less than 10% of the global health research resources were being applied to the health problems of developing countries, which accounted for over 90% of the world's health problems – an imbalance subsequently captured in the term the ‘10/90 gap’(35). Although awareness is increasing in LAMI countries about the importance of investing in mental health research to improve health care, combat poverty, decrease inequities and promote development (1, 8, 23, 35–40). The ‘10/90 gap’ remains visible in this field (3, 7, 35, 36, 41, 42).

To build a solid evidence base for advocacy and action in this area – and as a first step in their overall attempt to promote mental health research – the Global Forum for Health Research and the World Health Organization (WHO) launched a joint multi-country study to identify research capacity and priorities in mental and neurological health in LAMI countries (13).

### Aims of the study

The aim of this study was to describe the status of mental health research capacity of LAC countries in terms of number of indexed publications in Medline and PsycInfo databases, number of researchers, topic researched, and type of research and target population evaluated for the period from 1993 to 2003. Also, this study analyzed the scientific indicators of mental health output from LAC countries and universities available in ISI databases.

## Material and methods

A specific search strategy for mental health was developed to identify publications in which the first authors were affiliated with LAC institutions in Medline and PsycInfo databases covering the period between 1993 and 2003 (see Appendix). Indexed publications for countries with populations of more than 100 million, such as Brazil and Mexico, were selected and counted only for the years 1999–2003.

Publications were included if they dealt with mental disorders (anxiety and depression, psychoses, substance misuse, childhood disorders, learning disabilities, dementia, epilepsy, eating disorders, suicide, personality disorders, mental comorbidity with AIDS) or mental health issues. Publications were excluded if they were classified as letters, commentaries, based on animal studies, basic pharmacology and basic sciences (not related specifically with mental disorders), sexual dysfunction, medical complications of alcohol and drug use, child development and educational issues, neurological diseases not associated with mental disorders and psychoanalytic studies not addressing mental disorders.

The authors were selected from 30 LAC countries: Argentina, Belize, Bolivia, Brazil, Chile, Colombia, Costa Rica, Cuba, Dominica, the Dominican Republic, Ecuador, El Salvador, Grenada, Guatemala, Guyana, Haiti, Honduras, Jamaica, Mexico, Nicaragua, Panama, Paraguay, Peru, Saint Kitts and Nevis, Saint Lucia, Saint Vincent and the Grenadines, Suriname, Trinidad and Tobago, Uruguay and Venezuela.

Scientific indicators of LAC countries output in ‘Psychiatry and Psychology category’ were assessed using data from ‘ISI–- Essential Science Indicators’ database for the period January 1, 1995 and June 30, 2005. This database ranks all countries with publication output in ISI database by scientific indicators such as number of papers, citations and citations per paper, within a period of 10 years.

Scientific indicators of research institutions and universities from LAC in Psychiatry were obtained from the Atlas of Science database developed by the SCImago Research Group. This database provides information about scientific research in Ibero-American countries (Spanish- and Portuguese-speaking) using data extracted from the ISI database for the period 1990–2004. Data were available only for universities and research centers in Argentina, Brazil, Colombia, Chile, Mexico and Venezuela. The following scientific indicators were used: number of papers by country, institution, journal and author. The total number of ISI publications from 1990 to 2004 was used to rank respective universities and journals.

### Qualitative analysis of indexed-publications (Medline and PsycInfo) from LAC countries

All papers identified were classified according to 11 themes (childhood disorders, dementia, depression/anxiety, eating disorders, epilepsy, learning disorders, personality disorders, psychoses, substance misuse, suicide and others), type of research conducted (basic science, clinical research, clinical trials, epidemiological studies, health system research, economic and policy studies, methodology, social science and psychology studies, and others) and as well as by the mental health vulnerability of populations studied (children, disabled persons, the elderly, minorities, persons exposed to violence, the poor, prisoners, refugees, women and others).

### Quantitative analysis

Countries were compared according to the following variables: number of papers in three databases (ISI, Medline, PsycInfo); number of citations and citations per papers in ISI; number of first authors identified in Medline and PsycInfo; type of research; publication theme; and population studied. Universities were compared and ranked according to the total number of papers in Psychiatry (Atlas of Science database) from 1990 to 2004. Data from Atlas of Science database were used to rank the ISI-indexed journals in where publication from six LAC countries was more frequent for the period from 1990 to 2004.

spss software (version 11.5; SPSS Inc. Chicago, Illinois, USA) was used to analyze quantitative data. This study was approved by the Ethics Committee of Universidade Federal de São Paulo, Brazil and by the Universidad Cayetano Heredia, in Lima, Peru.

## Results

Table 1 shows that 1627 authors produced the 2397 articles indexed in Medline and PsycInfo databases between 1993 and 2003. Most of the authors identified in the combined Medline and PsycInfo database searches were from Brazil and Argentina (60% combined). Of the 30 LAC countries, 15 (50%) published 50 or less papers, 7 (23%) published 50 or more, and eight (27%) published none.

**Table 1:** Mental health research and publication output among LAC countries

				ISI (1995–2005)			Medline and Psycinfo (1993–2003)
LAC countries	Medline and Psycinfo (1993–2003) Number of papers *n* (%)	Medline (1993–2003) Number of papers *n*	Psycinfo (1993–2003) Number of papers *n*	Number of papers in the psychiatry and psychology categories *n* (%)	Number of citations	Citations per paper	Number of first authors
Argentina	665 (27.7)	374	291	187 (8.9)	968	5.18	455
Belize	0	0	0	–	–	–	0
Bolivia	14 (0.6)	4	10	–	–	–	13
Brazil*	792 (33.0)	441	351	586 (28.0)	3131	5.34	525
Chile	187 (7.8)	105	82	116 (5.6)	367	3.16	117
Colombia	114 (4.8)	45	69	118 (5.7)	298	2.53	69
Costa Rica	22 (0.9)	12	10	23 (1.1)	137	5.96	15
Cuba	42 (1.7)	27	15	30 (1.4)	128	4.27	33
Dominica	0	0	0	–	–	–	0
Dominican Republic	01 (0.1)	1	0	–	–	–	1
Ecuador	26 (1.1)	6	20		–	–	13
El Salvador	06 (0.2)	4	2	–	–	–	5
Grenada	01 (0.1)	0	1		–	–	1
Guatemala	11 (0.4)	4	7	–	–	–	7
Guyana	0	0	0	–	–	–	0
Haiti	03 (0.1)	1	2	–	–	–	3
Honduras	05 (0.2)	1	4	–	–	–	4
Jamaica	20 (0.8)	16	4	25 (1.2)	179	7.16	13
Mexico*	256 (10.7)	129	127	900 (43.0)	1840	2.04	193
Nicaragua	03 (0.1)	3	0	–	–		2
Panama	03 (0.1)	1	2	–	–	–	3
Paraguay	0	0	0	–	–	–	0
Peru	88 (3.7)	60	28	–	–	–	63
St Kitts and Nevis	0	0	0	–	–	–	0
St Lucia	0	0	0	–	–	–	0
St Vincent and the Grenadines	0	0	0	–	–	–	0
Suriname	0	0	0	–	–	–	0
Trinidad Tobago	14 (0.6)	12	2	12 (0.6)	88	7.33	11
Uruguay	25 (1.0)	2	23	–			24
Venezuela	99 (4.1)	42	57	88 (4.2)	356	4.05	57
Total	2397 (100.0)	1290	1107	2085 (100.0)	6620		1627

ISI, Institute for Scientific Information; LAC, Latin American and Caribbean.

* The number of papers from Brazil and Mexico in Medline and PsycInfo were counted only for the years 1999–2003.

Additionally, 2085 articles were identified in the ISI database (psychiatry/psychology section) from 1995 to 2005. Twenty countries (67%) were not listed in the ISI database. Six countries (Argentina, Brazil, Chile, Colombia, Mexico and Venezuela) accounted for 88% and 92% of LAC publications in the ISI and Medline/PscyInfo databases respectively. These countries were responsible for 92% of all citations of papers from LAC countries in ISI. Jamaica (9.3), Trinidad and Tobago (9.2), Mexico (8.4), Chile (7.3), Costa Rica (5.6) and Argentina (4.7) had the highest rate of ISI papers per million inhabitants. Of the 2397 publications found in the Medline and PsycInfo databases, 44% were in English, 41% in Spanish and 15% in Portuguese. Brazil accounted for 59% of the 720 papers published in English from 1999 to 2003.

Table 2 shows the 20 top institutions among six LAC countries in terms of the number of ISI-indexed publications in Psychiatry. The two leading institutions are located in the state of São Paulo, Brazil: the Universidade de São Paulo and the Universidade Federal de São Paulo. Among these 20 leading institutions, Brazilian universities accounted for 70% of publications outputs; Mexican universities, 23%; Argentinean universities, 5%; and Chilean universities 2.5%.

**Table 2:** Number of psychiatry and psychology papers in the ISI database produced in the 20 top Latin American universities with the most publications from 1990 to 2004

Country	Institution	Number of papers *n* (%)
Brazil	Universidade de São Paulo	753 (23.4)
Brazil	Universidade Federal de São Paulo	430 (13.3)
Mexico	Instituto Nacional de Psiquiatría Ramón de la Fuente Muñiz	417 (12.9)
Mexico	Universidad Nacional Autónoma de México	260 (8.0)
Brazil	Universidade Federal do Rio de Janeiro	204 (6.3)
Brazil	Universidade Estadual de Campinas	189 (5.9)
Brazil	Universidade Federal do Paraná	172 (5.3)
Brazil	Universidade Federal do Rio Grande do Sul	116 (3.6)
Brazil	Universidade Federal de Minas Gerais	98 (3.0)
Chile	Universidad de Chile	84 (2.6)
Argentina	Fundación para la Lucha contra las Enfermedades Neurológicas de la Infancia	83 (2.5)
Argentina	Universidad de Buenos Aires	80 (2.4)
Brazil	Universidade Federal Fluminense	58 (1.8)
Brazil	Universidade Estadual Paulista ‘Júlio de Mesquita Filho’	53 (1.6)
Mexico	Instituto Mexicano del Seguro Social	47 (1.4)
Brazil	Universidade Federal da Bahia	43 (1.4)
Mexico	Universidad Autónoma Metropolitana	37 (1.1)
Brazil	Universidade Federal de Santa Catarina	34 (1.0)
Brazil	Fundação Oswaldo Cruz	32 (0.9)
Brazil	Universidade Federal do Ceará	32 (0.9)
Total		3222 (100.0)

ISI, Institute for Scientific Information.

Source: Atlas of Science, SCImago Research group (http://www.atlasofscience.net) (accessed in December 2006).

Table 3 shows the distribution of papers in Psychiatry published in the 10 top ISI-indexed journals that carried most publications by authors from six LAC countries from 1990 to 2004. Of the 2426 papers published in these 10 journals during this period, 66% appeared in the Brazilian journal *Arquivos de Neuro-Psiquiatria* and 23% in the Mexican journal *Salud Mental*.

**Table 3:** Ten ISI-journals in which the leading Latin American countries published the highest number of psychiatry papers (from 1990 to 2004)

	Total	Brazil	Mexico	Argentina	Chile	Venezuela	Colombia
Arquivos de Neuro-Psiquiatria (Portuguese)	1157	1143	01	10	–	01	02
Salud Mental (Spanish)	564	01	546	03	08	01	05
Sleep (English)	139	110	26	02	10	–	01
Schizophrenia Research (English)	113	89	13	04	06	–	01
Psychopharmacology (English)	107	61	28	12	02	04	–
Biological Psychiatry (English)	99	51	26	11	08	03	–
Progress in Neuro-Psychopharmacology & Biological Psychiatry (English)	71	34	21	02	–	12	02
Journal of Neurology and Neurosurgery and Psychiatry (English)	69	25	–	31	12	–	01
Addiction (English)	57	38	09	–	08	01	–
European Neuropsychopharmacology (English)	50	38	10	–	–	02	–
Total	2426	1590	680	75	54	24	12

ISI, Institute for Scientific Information.

Source: Atlas of Science, SCImago Research group (http://www.atlasofscience.net) (accessed in December, 2006).

**Fig. 1:**
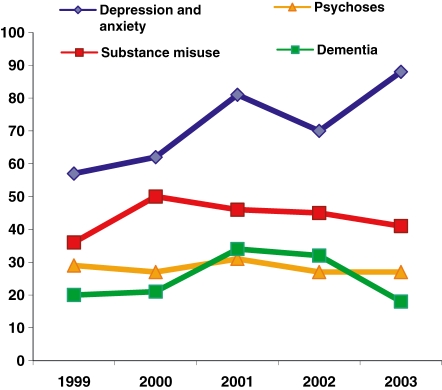
Classification of Medline and PsycInfo publications from the six leading counties (Argentina, Brazil, Chile, Colombia, Mexico and Venezuela) from 1999 to 2003 according to research topics.

The qualitative analysis of the 2397 LAC mental health publications in Medline and PsycInfo showed that most studies dealt with depression or anxiety (*n* = 476, 19.8%), substance misuse (*n* = 349; 14.6%), psychoses (*n* = 199; 8.3%), dementia (*n* = 192; 8.0%), childhood disorders (*n* = 148; 6.2%) and epilepsy (*n* = 149; 6.1%). Learning disabilities (*n* = 31; 1.3%), suicide (*n* = 54; 2.2%), eating disorders (*n* = 89; 3.7%) and personality disorders (*n* = 90; 3.7%) were the least represented themes. The annual number of studies on depression and anxiety almost doubled in the period 1999–2003, and there was a stable upward trend for studies on substance misuse and psychoses (Fig. 1).

**Fig. 2:**
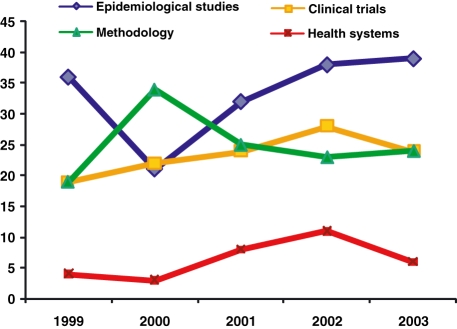
Classification of Medline and PsycInfo publications from the six leading counties (Argentina, Brazil, Colombia, Chile, Mexico and Venezuela) from 1999 to 2003 according to type of research.

Figure 2 shows that the annual number of epidemiological studies doubled from 2000 to 2003, while the number of clinical trials increased by 20%. Studies on clinical research (*n* = 761) and social sciences and psychology (*n* = 543) accounted for 54.5% of the publications, whereas clinical trials and health system and economic studies accounted for less than 8.5%. Thirty per cent of the publications focused on underprivileged or vulnerable populations. Studies on children (13.0%), women (3.7%) and the elderly (5.6%) accounted for 20% of overall publications, whereas studies involving people exposed to violence, the disabled, the poor, minorities, incarcerated people and refugees represented less than 1.5%.

## Discussion

This study revealed a significant imbalance in LAC mental health research output. It demonstrated that 90% of LAC mental health research is concentrated in the six countries in the region: Argentina, Brazil, Chile, Colombia, Mexico and Venezuela (22–33). Nevertheless, the highest rates of ISI publications per capita were found in smaller countries, such as Jamaica, and Trinidad and Tobago. This imbalance among LAC countries is even more evident when the number of publications indexed in three databases (Medline, PsycInfo and ISI) is compared: almost 1/4 of LAC countries had no scientific output according to Medline and PsycInfo database searches, and two-thirds had no publications indexed in the ISI database. These results suggest three different contexts of productivity and visibility in mental health research in LAC countries: a leading and competitive group of countries with production indexed in all three databases; an intermediate group, with a modest number of publications in one or more databases; and a third group of countries without any ISI publications and with very low (less than 50 publications) or non-existent publications in the other two databases.

This unfavorable scenario in mental health research in most LAC countries could be partially explained by their meager investment, where only 0.5% of their GDP goes to research and development (18, 22). Predictably, the leading position of countries such as Argentina, Brazil and Mexico is likely associated with the increase in their research budgets after 1990 (18, 23, 33). In 2003, Argentina and Brazil achieved a minimum acceptable level of 2.0% of their national health expenditure allocated for health research or research capacity strengthening, as recommended by the Commission on Health Research for Development (23). Although some studies have reported a correlation between the number of publications and the percentage of GDP spent on research and development (44), this sole parameter cannot be used to explain the continuous growth in research output of those countries because growth occurred even during periods when investment in research decreased (18, 22, 44).

Research in LAC countries is mostly funded by government agencies and 80% of the research output originates in state-managed universities (18, 25, 27, 29, 31). Although some isolated initiatives to fund health research based on mental health research priorities have been carried out, most research agencies do not generally allocate their funds according to priorities, quality or relevance of research projects (18, 27). Funding by external donors is also rarely aligned with the needs of LAMI countries (8, 9, 37, 40, 45). Although prioritizing mental health research was one of the 10 recommendations of the 2001 World Health Report (1), continuous efforts are still needed to adequately sensitize donors and policy-makers to the mental health demands of LAMI countries (1, 2, 8, 9, 11, 18, 36, 37, 43, 45, 46).

As well as increases in research funding, capacity building is a crucial step to develop mental health research in LAMI countries (3, 5, 7, 8, 35, 37, 43, 47, 48). The non-existent or low mental health research output in two-thirds of LAC countries may be understood in relation to several factors, such as the scarcity of mental health professionals, of graduate programmes and of psychiatrists involved in educational and research activities (21, 26, 49, 50). In this study, 12 countries (40%) had fewer than 20 researchers listed as first authors in Medline and PsycInfo publications, which suggests that mental health research in the region is predominantly conducted by a small pool of individuals, largely from Brazil and Argentina (18, 26).

During the past decade, greater investment in training researchers has boosted the development of mental health research in Brazil, Mexico and Chile (18, 22, 24–29, 51). Brazil and Mexico contribute 93% of the most productive institutions and graduate programmes in mental health research in LAC region. Graduate programmes tend to foster research culture and quality, and have a positive impact on the number of publications and the dissemination of research findings (25, 27, 28, 32, 44).

The development of a rigorous system for graduate programme evaluation was a crucial factor in achieving a leading position by Brazilian institutions. Over the past 5 years, Brazilian graduate programmes have been training about 50 doctoral students per year. Predictably, the number of Medline and ISI publications from the country doubled during this period (25, 27, 28). Brazil has established a stable training funding system – the so-called ‘sandwich scholarship’– whereby several programmes are supported to train graduate students abroad. This fellowship scheme covers expenses for students to spend 1 year abroad as part of their training, although some constraints remain in terms of salaries and tuitions fees (47, 48).

In parallel with unsatisfactory conditions for increasing research output by LAC researchers, the ‘brain drain’ phenomenon, especially for junior researchers, has grown in some countries, mainly because of a lack of national policies to ensure support for research and promotion of researchers’ careers (22, 51). In LAC countries, research budgets are not allocated to finance researchers’ salaries and a large percentage of researchers work as volunteers (52). To help stall the brain drain and to enhance research efforts, the Pan American Health Organization (PAHO) and the United States National Institute of Mental Health (NIMH), have been promoting a number of conferences in the region, such the one held recently in Mexico (45). The key strategies for improving research capacity raised in these conferences were: the critical need for training mental health professionals in research methods and scientific writing, making mental health research attractive to young researchers, promoting strategies for acquiring research grants, developing and sustaining researcher's careers, increasing the level of networking among research teams, enhancing research dissemination and fostering dialogue between research teams and policy-makers (45).

Strengthening health and psychiatric journals in LAMI countries may also be an important strategy to inform policy-makers about mental health demands, to promote research development and to enhance research dissemination (2, 6, 8, 39, 53). The increase in the number of citations of LAC publications, mainly observed in Argentina, Brazil, Chile and Mexico could be considered as an indicator of increasing international visibility of mental health research of such countries (27, 32). In fact, a recent study showed no significant differences in the number of ISI citations or downloads of articles published in Acta Psychiatrica Scandinavica between those articles from high-income countries and from low-and-middle-income countries (54). Nevertheless, it is important to highlight current constraints for disseminating research findings, such as language barriers, lack of experience in writing papers to international standards, low manuscript submission rates, possible acceptance biases and the low number of LAC mental health journals (3, 5–7, 10, 27, 39, 53–56).

The leading positions of Brazil and Mexico could also be explained by the fact that only two Latin American psychiatric journals (*Arquivos de Neuro-Psiquiatria* from Brazil and *Salud Mental* from Mexico) were indexed in the ISI database during the period analyzed in this study. However, the absence of Brazilian authors in the Mexican journal and vice-versa makes evident a high degree of indigenous bias. These two journals and the *Revista Brasileira de Psiquiatria*, the Brazilian journal most recently indexed in the ISI database, might consider playing a more active role in the representation of LAC countries in editorial boards and, in promoting research dissemination from the region (5, 6).

In LAMI countries, scientific evidence of socio-economic and cultural conditions that influence mental disorders and their burden is still scarce (1, 8, 13, 15, 27, 31, 32, 57–60). Despite the high prevalence of infectious diseases in these countries, non-communicable diseases account for more than 70% of the mortality and the global burden of disease (14). Although cardiovascular diseases are responsible for the highest rates of mortality, mental health and injuries are responsible for 36% of the total disability-adjusted life years in LAC countries (14).

The results of this study revealed a predominance of publications related to the three most burdensome (burden mental disorders – depression, alcohol disorder and schizophrenia) and a paucity of studies on suicide, childhood disorders, learning disabilities, eating and personality disorders. Moreover, studies among populations at risk for mental disorders, such as women, children and people exposed to violence, were under represented in the LAC region.

The quality and relevance of the mental health studies conducted in LAC countries are key factors influencing the ultimate dissemination of findings in the international literature, as well the provision of scientific evidence to decision-makers (6, 7, 27, 53, 55, 61). A critical mass of researchers competent in conducting studies in basic science, neuroscience, epidemiology and health economics, and health systems remains to be established. In the past decade, however, the number of clinical trials in the region rose 10-fold as a result of the growing interest by funding sponsors, especially pharmaceutical industries (62, 63). The number of epidemiological and clinical studies has also increased in recent years, but epidemiological investigations acceptable standards, including longitudinal design are largely absent (30). Moreover, there are very few studies assessing the cost-effectiveness of therapeutic interventions, the performance of mental health systems and interventions to reduce the burden of disease, especially among the poor (12, 64, 65).

Finally, the development of mental health research varies significantly across LAC countries in relation to research capacity, quality, relevance, funding and dissemination despite similar economic and cultural features. The six leading LAC countries should consider extending mental health research training to their neighbors through bilateral or multilateral projects, possibly, under the auspices of PAHO. Furthermore, researchers should be more aware of their social and political roles and responsibilities in communicating research priorities to policy-makers, donors and the media. Efforts should be concentrated on enhancing and sustaining funding, focusing on capacity building, promoting research careers, prioritizing funding for studies on mental health systems and burdensome conditions, sensitizing policy-makers to the burden of mental disorders, and ultimately improving scientific research and its dissemination.

## Appendix

Search ‘Psychiatry and Psychology Category’[MeSH] OR ‘Epilepsy’[MeSH] OR ‘Dementia’[MeSH] OR ‘Sleep Disorders’[MeSH] OR ‘Neuroleptic Malignant Syndrome’[MeSH] OR ‘AIDS Dementia Complex’[MeSH] OR ‘Dual Diagnosis’[MSH] OR ‘mental health’ OR ‘learning disabilities’ OR suicide OR suicide[MeSH Subheading] OR psych* Limits: Human, MEDLINE.
